# Independent associations of serum calcium with or without albumin adjustment and serum phosphorus with nonalcoholic fatty liver disease: results from NHANES 1999-2018

**DOI:** 10.3389/fendo.2024.1323990

**Published:** 2024-03-05

**Authors:** Haolong Qi, Bin Wang, Lei Zhu

**Affiliations:** Department of Hepatobiliary Surgery, Renmin Hospital of Wuhan University, Wuhan, China

**Keywords:** calcium, NHANES, albumin, phosphorus, nonalcoholic fatty liver disease

## Abstract

**Background:**

The associations of serum calcium and phosphorus with nonalcoholic fatty liver disease (NAFLD) remain unclear. In addition, there may be an effect of albumin correction on the association between serum calcium and NAFLD. We aimed to explore these relationships in the National Health and Nutrition Examination Survey (NHANES).

**Methods:**

Eligible adult individuals from NHANES 1999-2018 were recruited for the study. We explored the associations of serum calcium, albumin-adjusted serum calcium, and serum phosphorus with NAFLD in multivariable-adjusted regression models. In addition, restricted cubic spline (RCS), stratified analysis, and multiple sensitivity analyses were used for further elaboration.

**Results:**

The study sample consisted of 20,900 participants, with an observed NAFLD prevalence of 44.65%. Fully adjusted models indicated that serum calcium was inversely associated with NAFLD (odds ratio [OR] and 95% confidence interval [CI] = 0.70 (0.62, 0.78), p<0.0001), whereas albumin-adjusted serum calcium was positively associated with NAFLD (OR and 95% CI=1.59 (1.41, 1.79), p<0.0001). RCS modeling indicated that serum calcium without and with albumin adjustment was linearly(p nonlinear = 0.083) and nonlinearly (p nonlinear < 0.0001) associated with NAFLD, respectively, whereas serum phosphorus showed a U-shaped relationship with NAFLD(p nonlinear < 0.0001). Gender is a significant influence in all associations, and other variables may also have an effect. Sensitivity analyses indicated that these associations were independent of additional significant confounders.

**Conclusion:**

Serum calcium and phosphorus were significantly associated with the development of NAFLD. These findings suggest the potential clinical significance of serum calcium/phosphorus and albumin levels in individuals at high risk for NAFLD. Our study supports the potential role of serum calcium/phosphorus homeostasis in the pathophysiology of NAFLD and could serve as NAFLD-related biomarkers.

## Introduction

Nonalcoholic fatty liver disease (NAFLD) refers to a pathological condition in which intracellular lipid accumulation in the liver exceeds 5% in the absence of secondary chronic liver injury factors, which can range from simple hepatic steatosis to the more severe form of nonalcoholic steatohepatitis (NASH), and in a small percentage of patients, can progress to cirrhosis and hepatocellular carcinoma (HCC) (1, 2). NAFLD is currently the most common chronic liver disease in the world, and its prevalence has risen considerably compared to past decades. A recent large meta-analysis suggests that one in three adults is likely to suffer from NAFLD and it will continue to increase at an alarming rate in the future (3). NAFLD is currently the leading cause of liver transplantation and HCC, resulting in a substantial public health burden each year (4, 5). In addition, NAFLD is a multisystemic disease whose prevalence generally parallels that of obesity and type 2 diabetes mellitus, and is associated with the development of a variety of extrahepatic disorders, such as chronic kidney disease, cardiovascular disease (CVD), multiple cancers, and neuropsychiatric disorders (6). Despite the numerous NAFLD-related clinical trials currently being conducted, there are still no FDA-approved pharmacotherapy for NAFLD/NASH (7). Therefore, identifying risk factors associated with the development of NAFLD has important clinical relevance in improving our understanding of the pathogenesis of NAFLD as well as risk stratification of populations.

Calcium is an indispensable macronutrient for the human body and is known as the “life element”. Calcium plays a pivotal role in human health and disease and is essential for the normal functioning of multiple systems such as the cardiovascular system, muscles, nerves, and bones (8–10). Ninety-nine percent of the calcium in the human body is distributed in the bones and teeth, with the remaining 1% in the blood, intercellular fluid, and soft tissues (11). Serum total calcium is divided into bound calcium and ionized calcium (each accounting for about half of serum total calcium), with albumin being the major calcium-binding protein (12). Blood calcium is mainly derived from absorption from the digestive tract and from calcium in the bones, and its levels are also regulated by several hormones. Serum calcium and serum phosphorus have a close correlation; normally the calcium-phosphorus product is maintained within a constant range, and its abnormality is associated with a variety of diseases (13, 14).

The association between serum calcium and phosphorus and NAFLD remains poorly studied. Currently, only three observational studies, all from Asia (China and Korea), have explored the relationship between serum calcium and NAFLD, and the conclusions were inconsistent. Two case-control studies showed that serum calcium was negatively associated with NAFLD (15, 16), while another study showed that serum calcium and phosphorus were significantly and positively associated with NAFLD (17). Of these studies, only the study by Shin et al. (17) used albumin-corrected serum calcium as an adjustment for serum calcium. In addition, addressing these associations could help to elucidate whether serum calcium/phosphorus may serve as a potential biomarker for the development of NAFLD and provide clinical evidence regarding the necessity of focusing on serum calcium/phosphorus homeostasis in at-risk populations. We here explore these relationships using a nationwide population-based survey, the National Health and Nutrition Examination Surveys (NHANES). To elucidate these correlations, we explored the association of serum total calcium (with or without albumin adjustment) and serum phosphorus with NAFLD. These results may help shed light on these controversies and provide useful information for clinicians.

## Methods

### Study population

NHANES is a cross-sectional, publicly accessible survey designed to investigate the health and nutritional status of noninstitutionalized populations in the US, which is a major program of the National Center for Health Statistics (NCHS). NHANES is a complexly designed, multistage, probability-sampling, population-based survey that includes interviews and various types of examination data and has been conducted in biennial cycles since 1999. Therefore, NHANES is the primary database used to explore the epidemiology and associated risk factors of diseases, and numerous important studies have been published to date, which have greatly improved the understanding of these conditions. The NHANES was reviewed and approved by the NCHS Ethics Review Board (ERB) and therefore do not require additional ethical review consent. In addition, written consent was obtained from all participants in this survey.

**Figure 1:**
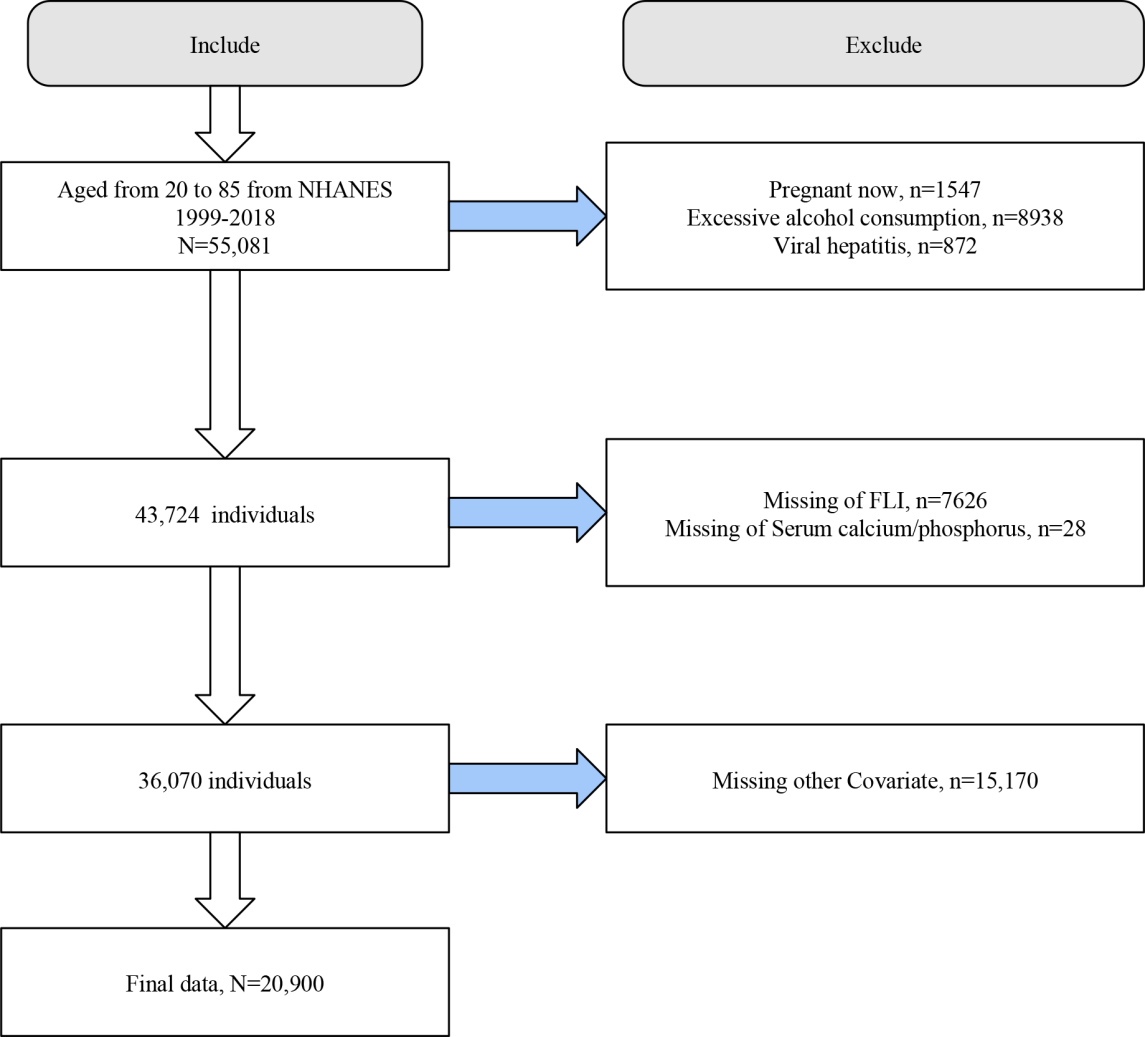
Flowchart of the study population selection process.

We initially included 55,081 individuals aged 20-85 years from NHANES 1999-2018. We first excluded participants with pregnancy (n=1547), excessive alcohol consumption (n=8938), and viral hepatitis (n=872). Next, we excluded participants who lacked information on FLI (n=7626), serum calcium/phosphorus (n=28), and covariates (n=15170). Finally, we included 20,900 eligible participants for analysis (**Figure 1**).

### Evaluation of serum calcium and phosphorus

Serum total calcium and phosphorus levels were obtained by measurement in blood samples collected and processed at a mobile screening center. These assays are part of routine biochemical analysis and are performed with a Beckman Synchron LX20. The LX20 system uses indirect (or diluted) ISE methodology to measure calcium concentration and a timed-rate method to determine the concentration of phosphorus in serum. For albumin-corrected serum calcium, the formula is as follows: albumin-corrected serum calcium (mg/dL) = serum total calcium (mg/dL) + 0.8 * (4(g/dL) - serum albumin (g/dL)) (18). In addition, to account for the heterogeneity of the serum albumin reference values, we used another corrected formula for the sensitivity analysis: albumin-corrected serum calcium (mg/dL) = serum total calcium (mg/dL) + 0.8 * (4.4(g/dL) - serum albumin (g/dL)). In NHANES, biochromatic digital endpoint method was used to measure the albumin concentration.

### Definition of NAFLD

The NHANES survey only introduced imaging tools (transient elastography) as a diagnostic method for hepatic steatosis for the first time in 2017-2018. To allow for the comprehensiveness of the survey cycle and the large sample size, we used the NHANES 1999-2018 samples in this study and used two noninvasive markers for the diagnosis of NAFLD, which have been shown to have good accuracy. We used a well-validated noninvasive algorithm, the Fatty Liver Index (FLI), as a surrogate for NAFLD. FLI was derived from serum triglycerides (TG), body mass index (BMI), waist circumference (WC), and serum gamma-glutamyl transpeptidase (GGT). FLI = (e^0.953*loge (TG) + 0.139*BMI + 0.718*loge (GGT) + 0.053*WC - 15.745^)/(1 + e^0.953*loge (TG) + 0.139*BMI + 0.718*loge (GGT) + 0.053*WC - 15.745^) × 100 (19). FLI ≥ 60 and exclusion of other chronic liver diseases was considered to have presumptive NAFLD. We performed sensitivity analyses using another commonly used surrogate for NAFLD, the USFLI, to verify the consistency of the results. The USFLI was developed for the multiethnic cohort based on NHANES, which has improved predictive accuracy for NAFLD compared to the FLI based on age, ethnicity, WC, GGT, fasting insulin, and fasting glucose. USFLI = (e^-0.8073 × non-Hispanic black + 0.3458 × Mexican American + 0.0093 × age + 0.6151 × ln (GGT) + 0.0249 × WC + 1.1792 × ln (insulin) + 0.8242 × ln (glucose) -14.7812^)/(1 + e^−0.8073 × non-Hispanic black + 0.3458 × Mexican American + 0.0093 × age + 0.6151 × ln (GGT) + 0.0249 × WC + 1.1792 × ln (insulin) + 0.8242 × ln (glucose) -14.7812^) × 100 (20). A USFLI ≥ 30 was considered to have NAFLD.

### Covariates

We selected several important potential covariates based on previous studies, including age, gender, ethnicity, education level, marital status, family income to poverty (PIR), smoking, diabetes, hypertension, CVD, estimated glomerular filtration rate (eGFR), physical activity, and dietary calcium/phosphorus intake. Smoking status was obtained by self-report in the questionnaire and categorized as non-smoking (less than 100 lifetime cigarettes), former smoking (smoked more than 100 cigarettes in lifetime but not smoking now), and current smoking (>100 cigarettes in lifetime and currently smoking). Diabetes was diagnosed based on self-reported history of diabetes (diagnosed by a physician), a blood glucose/glucose tolerance test that meets the American Diabetes Association’s criteria or being on anti-diabetic medication. Hypertension was indicated by either of the following: self-reported hypertension, blood pressure ≥130/85 mmHg, or use of antihypertensive medications. The diagnosis of CVD was determined by self-report on the NHANES questionnaire. eGFR was calculated based on the Chronic Kidney Disease Epidemiology Collaboration (CKD-EPI) equation (21). Physical activity was measured in metabolic equivalents [MET] according to the questionnaire and MET ≥ 600 min/week was considered physically active (22). Dietary calcium/phosphorus intake was calculated based on the average of two dietary recall interviews in NHANES.

### Statistical analysis

All analyses were conducted using EmpowerStats (X&Y Solutions, Inc., Boston, MA) and R software 4.2.3. Considering the complex study design of NHANES, as well as to make our sample representative of the overall US population, we weighted our analyses accordingly (23). In the baseline analysis, continuous variables were expressed using mean ± standard error, while categorical variables were described using number (percentage). Student’s t-test was used for continuous variables and chi-square test for categorical variables.

We constructed several multivariate adjusted logistic regression models. The crude model did not adjust for any covariates. Model 1 adjusted for age, sex, race, education, PIR, and marital status; model 2 additionally adjusted for smoking, diabetes, hypertension, CVD, and physical activity in addition to model 1; and model 3 was a fully adjusted model with additional adjustments for eGFR and dietary calcium/phosphorus intake in addition to model 2.

To explore potential nonlinear associations between serum calcium and phosphorus and NAFLD, we performed restricted cubic spline curve (RCS) analysis. We used stratified analyses to explore the consistency of the results. Several sensitivity analyses were performed to examine the robustness and stability of the results. First, we used USFLI as a diagnostic indicator for NAFLD for analysis. Second, we further adjusted serum vitamin D or parathyroid hormone (PTH) levels under the previous fully adjusted model. Data for vitamin D (25(OH)D) were accessible in NHANES 2001-2018, and we analyzed and standardized them based on previous studies (24). Information on PTH was only available in NHANES 2003-2006, which was obtained from the Elecsys 1010 analyzer. Next, we explored whether serum calcium and phosphorus were still associated with NAFLD in a population with normal bone mineral density (BMD) at the femoral neck. Normal BMD was defined as a t-score (compared with the standard deviation of BMD in healthy young people of the same sex) > -1 (25). Finally, we performed a sensitivity analysis for serum calcium correction using another serum albumin reference value. In 2023, an international panel of experts updated the nomenclature of NAFLD as steatotic liver disease (SLD) (26). Therefore, we additionally explored here whether serum calcium/phosphorus is associated with SLD and metabolic dysfunction-associated steatotic liver disease (MASLD). We included participants from NHANES 2017-2020 and diagnosed SLD and MASLD according to the recently published official consensus, in which hepatic steatosis was confirmed using a CAP of >248 dB/m. In all analyses, p < 0.05 was considered statistically significant.

## Results

### Baseline characteristics

We included a total of 20,900 participants (mean age 48.05 years, 48.72% male), and there were 9,332 FLI-NAFLD subjects, corresponding to a prevalence of 44.65%. Participants with NAFLD had higher age, proportion of men, proportion of smokers (former and current), and higher prevalence of diabetes, hypertension, and CVD compared to those without NAFLD. However, the NAFLD population had lower PIR, eGFR, albumin levels, and lower proportions of non-Hispanic White, single, and education attainment above high school. Notably, serum calcium and phosphorus levels were lower in patients with NAFLD, whereas in contrast, albumin-adjusted serum calcium levels and dietary phosphorus intake were higher (**Table 1**).

**Table 1:** Baseline characteristics grouped according to NAFLD.

Variable	Total (n=20,900)	Non-NAFLD (n=11,568)	NAFLD (n=9,332)	P value
Age, year	48.05 ± 0.23	46.25 ± 0.28	50.44 ± 0.25	< 0.0001
PIR	3.29 ± 0.03	3.35 ± 0.03	3.20 ± 0.03	< 0.0001
Physical activity, Met/min-week	3017.59 ± 65.05	2979.82 ± 72.07	3067.97 ± 83.37	0.3
eGFR, ml/min/1.73m²	92.53 ± 0.31	94.05 ± 0.37	90.51 ± 0.33	< 0.0001
Calcium intake, mg/day	956.70 ± 6.04	957.54 ± 7.72	955.58 ± 7.44	0.83
Phosphorus intake, mg/day	1380.36 ± 6.55	1361.29 ± 8.32	1405.80 ± 8.46	< 0.0001
Serum phosphorus, mg/dl	3.70 ± 0.01	3.72 ± 0.01	3.68 ± 0.01	< 0.0001
Serum total calcium, mg/dl	9.44 ± 0.01	9.44 ± 0.01	9.42 ± 0.01	0.003
Albumin-corrected serum total calcium, mg/dl	9.19 ± 0.01	9.17 ± 0.01	9.23 ± 0.01	< 0.0001
Albumin, g/dl	4.30 ± 0.00	4.35 ± 0.00	4.24 ± 0.01	< 0.0001
Sex				< 0.0001
male	10416 (48.72)	5212 (41.92)	5204 (57.78)	
female	10484 (51.28)	6356 (58.08)	4128 (42.22)	
Race				< 0.0001
Mexican American	2917 (5.73)	1371 (4.91)	1546 (6.81)	
Non-Hispanic Black	4062 (9.40)	2157 (8.94)	1905 (10.02)	
Non-Hispanic White	10581 (74.38)	5976 (74.88)	4605 (73.70)	
Other Hispanic	1449 (4.38)	778 (4.32)	671 (4.46)	
Other Race	1891 (6.12)	1286 (6.95)	605 (5.01)	
Marital Status				< 0.0001
non-single	13542 (69.31)	7326 (67.69)	6216 (71.48)	
single	7358 (30.69)	4242 (32.31)	3116 (28.52)	
Education				< 0.0001
<high school	1658 (3.59)	811 (3.29)	847 (3.98)	
high school	6884 (29.79)	3512 (26.80)	3372 (33.79)	
>high school	12358 (66.62)	7245 (69.91)	5113 (62.23)	
Smoking				< 0.0001
never	12267 (59.35)	7201 (62.60)	5066 (55.01)	
former	5534 (26.24)	2634 (22.85)	2900 (30.77)	
now	3099 (14.41)	1733 (14.55)	1366 (14.23)	
Diabetes				< 0.0001
Yes	3208 (11.21)	911 (4.94)	2297 (19.58)	
Hypertension				< 0.0001
Yes	8542 (35.59)	3534 (24.94)	5008 (49.80)	
CVD				< 0.0001
Yes	2049 (7.56)	853 (5.28)	1196 (10.60)	

NAFLD, nonalcoholic fatty liver disease; PIR, family income to poverty; eGFR, estimated glomerular filtration rate. Continuous variables were expressed using mean ± standard error, while categorical variables were described using number (percentage).

In addition, we conducted baseline analyses of the included population based on quartile distributions of serum calcium, albumin-corrected serum calcium, and serum phosphorus. **Supplementary Table S1** described the range of interquartile values for each variable, and **Supplementary Tables S2**–**S4** summarized the baseline information based on these exposure quartiles. We found that the prevalence of NAFLD decreased significantly with increasing quartiles of serum calcium and phosphorus (both p < 0.05), whereas albumin-adjusted serum calcium showed an opposite trend to the prevalence of NAFLD.

### Multivariate adjusted regression analysis

We explored whether serum calcium (with or without albumin adjustment) and phosphorus were independently associated with the development of NAFLD through multiple multivariable-adjusted models. Serum calcium was inversely associated with the prevalence of NAFLD after adjusting for all confounding variables (odds ratio [OR] and 95% confidence interval [CI] = 0.70 (0.62, 0.78), p<0.0001). We observed a significant dose-response correlation between serum calcium and NAFLD (p for trend <0.0001), with serum calcium in Q3 and Q4 associated with 18% and 31% lower prevalence of NAFLD compared to Q1, respectively. However, surprisingly, albumin-adjusted serum calcium was positively associated with NAFLD (OR and 95% CI=1.59 (1.41, 1.79), p<0.0001). A similar dose-response correlation was observed (p for trend <0.0001). Serum phosphorus as a categorical variable was negatively associated with NAFLD, and serum phosphorus in Q2, Q3, and Q4 compared with Q1 was associated with 18%, 22%, and 11% lower risk of NAFLD. However, there was no significant correlation when used as a continuous variable, suggesting a possible nonlinear correlation between serum phosphorus and NAFLD (**Table 2**).

**Table 2:** Association of serum calcium, albumin-adjusted serum calcium, and serum phosphorus with NAFLD.

	Crude Model OR (95%CI) P-value	Model 1 OR (95%CI) P-value	Model 2 OR (95%CI) P-value	Model 3 OR (95%CI) P-value
**Serum calcium**	0.85 (0.76, 0.94) 0.0031	0.78 (0.69, 0.87) <0.0001	0.69 (0.62, 0.78) <0.0001	0.70 (0.62, 0.78) **<0.0001**
Serum calcium quartile				
Q1	Ref.	Ref.	Ref.	Ref.
Q2	0.93 (0.81, 1.05) 0.2316	0.92 (0.80, 1.04) 0.1941	0.90 (0.79, 1.02) 0.1104	0.90 (0.79, 1.02) 0.1144
Q3	0.91 (0.81, 1.02) 0.1168	0.86 (0.76, 0.98) 0.0198	0.82 (0.73, 0.93) 0.0023	0.82 (0.73, 0.93) **0.0026**
Q4	0.83 (0.74, 0.94) 0.0050	0.77 (0.67, 0.87) 0.0001	0.69 (0.60, 0.79) <0.0001	0.69 (0.61, 0.79) **<0.0001**
P for trend	0.0043	<0.0001	<0.0001	**<0.0001**
**Albumin-adjusted serum calcium**	1.78 (1.60, 1.98) <0.0001	1.81 (1.61, 2.03) <0.0001	1.57 (1.40, 1.77) <0.0001	1.59 (1.41, 1.79) **<0.0001**
Albumin-adjusted serum calcium quartile				
Q1	Ref.	Ref.	Ref.	Ref.
Q2	1.25 (1.12, 1.39) 0.0001	1.26 (1.13, 1.41) 0.0001	1.24 (1.11, 1.39) 0.0003	1.25 (1.11, 1.40) **0.0002**
Q3	1.44 (1.30, 1.59) <0.0001	1.46 (1.31, 1.62) <0.0001	1.42 (1.28, 1.57) <0.0001	1.43 (1.29, 1.58) **<0.0001**
Q4	1.65 (1.48, 1.84) <0.0001	1.68 (1.50, 1.89) <0.0001	1.48 (1.32, 1.67) <0.0001	1.50 (1.33, 1.68) **<0.0001**
P for trend	<0.0001	<0.0001	<0.0001	**<0.0001**
**Serum phosphorus**	0.86 (0.81, 0.91) <0.0001	0.97 (0.91, 1.02) 0.2324	0.95 (0.90, 1.01) 0.1108	0.95 (0.90, 1.01) 0.1178
Serum phosphorus quartile				
Q1	Ref.	Ref.	Ref.	Ref.
Q2	0.79 (0.72, 0.87) <0.0001	0.84 (0.76, 0.92) 0.0005	0.82 (0.74, 0.91) 0.0003	0.82 (0.73, 0.91) **0.0003**
Q3	0.69 (0.63, 0.77) <0.0001	0.78 (0.70, 0.87) <0.0001	0.78 (0.70, 0.87) <0.0001	0.78 (0.70, 0.87) **<0.0001**
Q4	0.76 (0.70, 0.83) <0.0001	0.91 (0.83, 1.00) 0.0460	0.89 (0.80, 0.98) 0.0177	0.89 (0.80, 0.98) **0.0187**
P for trend	<0.0001	0.1233	0.0778	0.0805

NAFLD, nonalcoholic fatty liver disease; OR, odds ratio; eGFR, estimated glomerular filtration rate. The crude model did not adjust for any covariates. Model 1 adjusted for age, sex, race, education, PIR, and marital status; model 2 additionally adjusted for smoking, diabetes, hypertension, CVD, and physical activity in addition to model 1; and model 3 additionally adjusted for eGFR and dietary calcium/phosphorus intake in addition to model 2.

The bold data represents statistically significant data in the fully adjusted model (p<0.05).

### RCS model

**Figure 2:**
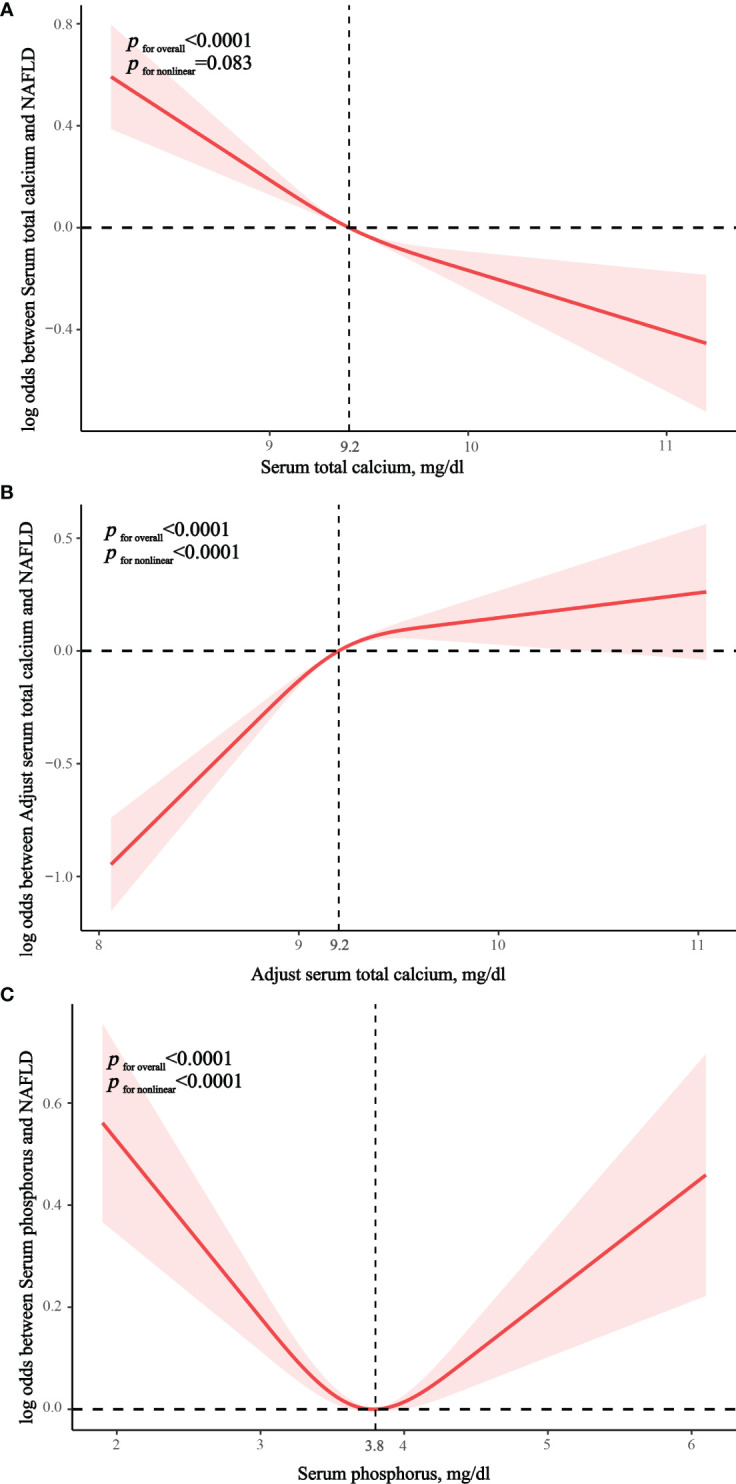
Restricted cubic spline exploring nonlinear relationships. **(A)** The correlation between serum total calcium and NAFLD. **(B)** The correlation between albumin-adjusted serum calcium and NAFLD. **(C)** The correlation between serum phosphorus and NAFLD.

In the fully adjusted RCS model, we found no nonlinear correlation between serum calcium and NAFLD (p nonlinear = 0.083), suggesting that serum calcium was linearly associated with the prevalence of NAFLD. (**Figure 2A**) There was significant nonlinear correlation between albumin-adjusted serum calcium and serum phosphorus and NAFLD (both p nonlinear < 0.0001) and serum phosphorus showed a U-shaped correlation with NAFLD. (**Figures 2B, C**) Piecewise regression analysis showed that albumin-adjusted serum calcium flattened out in relation to NAFLD after 9.2 mg/dL, whereas serum phosphorus lost its association with NAFLD after 3.8 mg/dL (**Supplementary Table S5**).

### Stratified analysis

**Figure 3:**
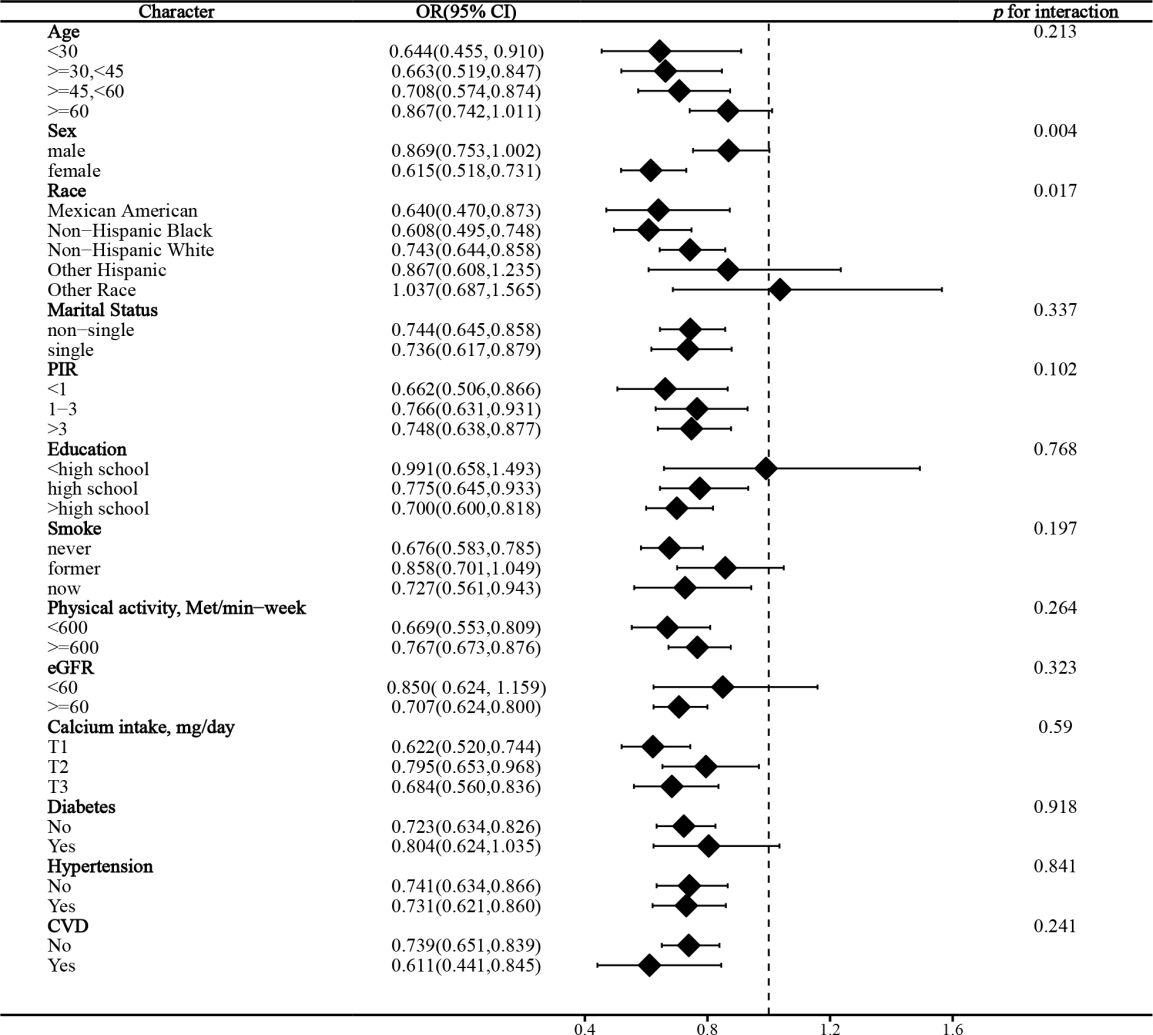
Stratified analysis of serum calcium in relation to NAFLD.

**Figure 4:**
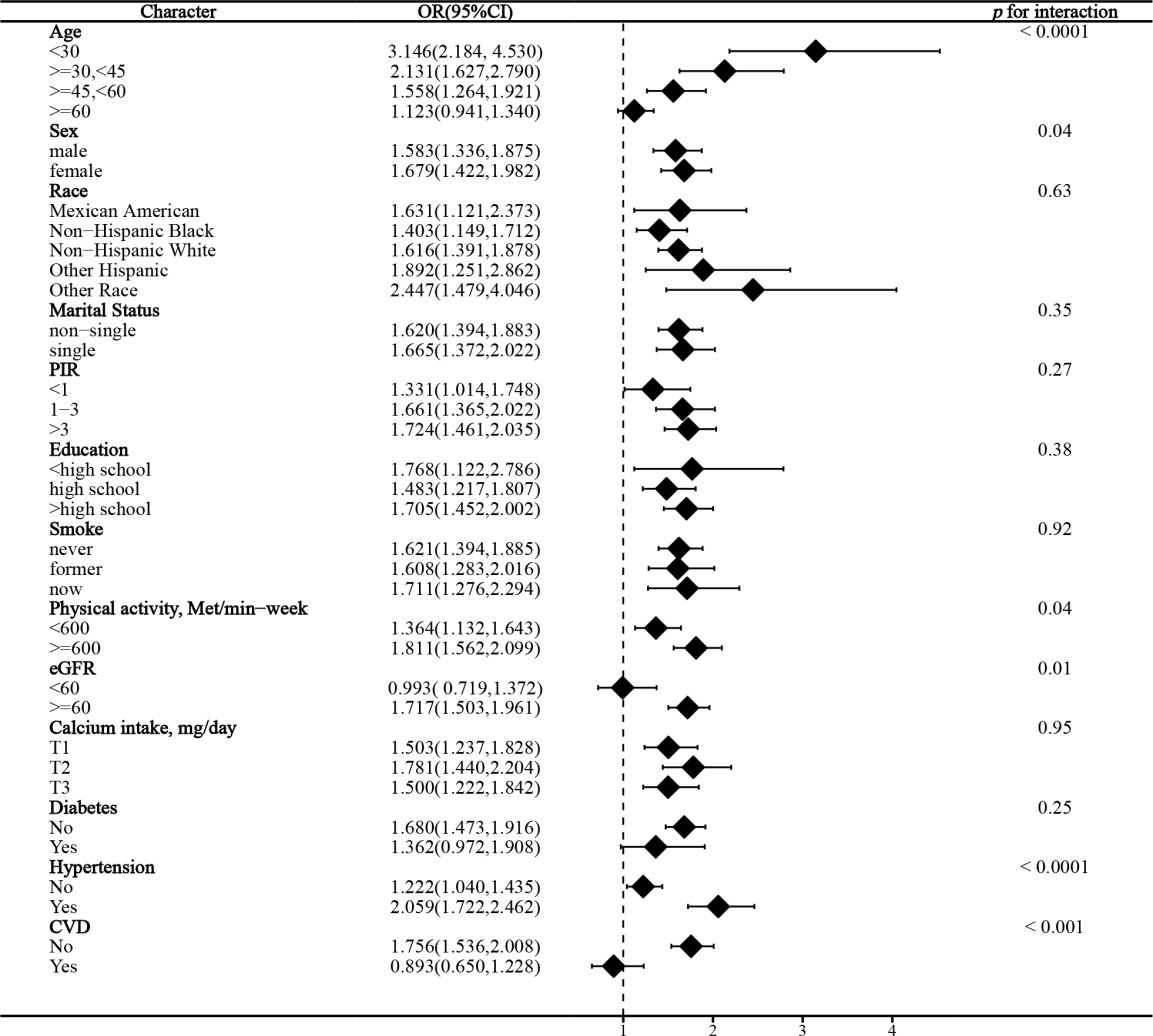
Stratified analysis of albumin-adjusted serum calcium in relation to NAFLD.

**Figure 5:**
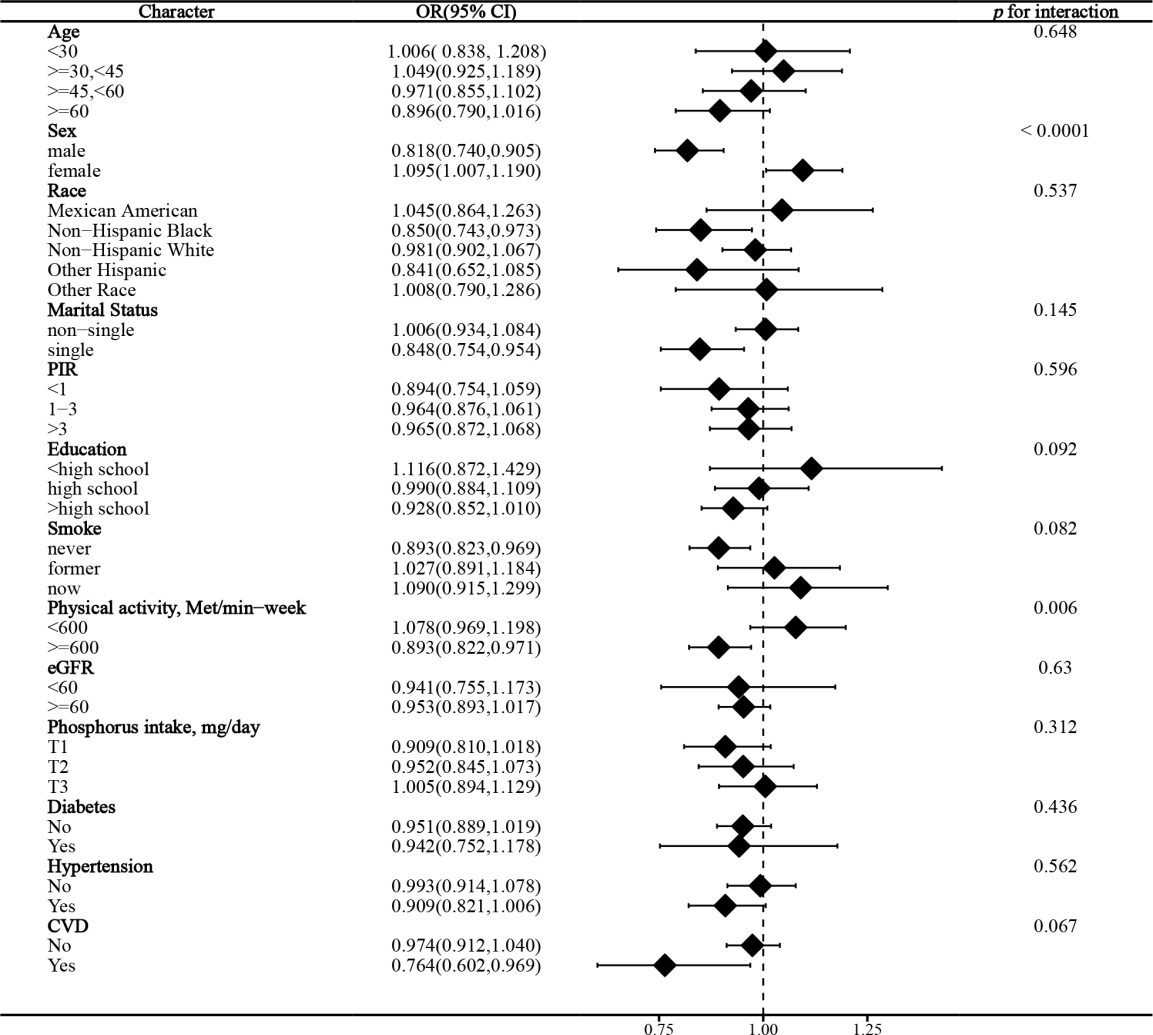
Stratified analysis of serum phosphorus in relation to NAFLD.

In the stratified analysis of the association between serum calcium and NAFLD, we found that gender and race significantly influenced the relationship (p for interaction =0.004 and 0.017, respectively). (**Figure 3**) We found that age, gender, physical activity, eGFR, hypertension, and CVD were significant influencers of the albumin-adjusted serum calcium-NAFLD relationship (p for interaction all <0.05). (**Figure 4**) Finally, gender and physical activity also significantly influenced the association of serum phosphorus with NAFLD (**Figure 5**).

### Sensitivity analysis

We first performed a sensitivity analysis using USFLI to diagnose NAFLD. We found that serum calcium with and without albumin adjustment and serum phosphorus were similarly associated with NAFLD. (**Supplementary Table S6**) Next, we additionally adjusted for vitamin D based on model 3 and obtained similar results. (**Supplementary Table S7**) Similar results were observed for the additional adjustment for PTH based on model 3. (**Supplementary Table S8**) Finally, we still noted similar results in a population with normal BMD, i.e., serum calcium and phosphorus remained inversely associated with NAFLD, whereas albumin-adjusted serum calcium was positively associated with NAFLD. (**Supplementary Table S9**) These results suggested that serum calcium with or without albumin-adjusted and serum phosphorus were associated with NAFLD independently of these important confounders, demonstrating the robustness of our results. Finally, we obtained similar results using another serum albumin reference value (4.4 g/dL) for calcium correction, indicating the stability of our results. (**Supplementary Table S10**) We found that only albumin-adjusted serum calcium was significantly positively associated with MASLD (OR and 95% CI = 1.60 (1.13, 2.26), p = 0.0025) and SLD (1.39 (1.12,1.74), p = 0.0067) in the fully adjusted model, and that higher levels of corrected serum calcium were associated with higher odds of MASLD (p for trend = 0.0437) and SLD (p for trend = 0.0098) (**Supplementary Table S11**).

## Discussion

To our knowledge, this is the first time that the association of serum calcium with and without adjustment for albumin and serum phosphorus with NAFLD has been explored in a US representative population-based study. We found that serum total calcium was negatively correlated with NAFLD, whereas, interestingly, albumin-adjusted serum calcium was positively associated with NAFLD. We revealed that serum calcium showed a linear correlation with NAFLD, whereas albumin-adjusted serum calcium was nonlinearly associated with NAFLD. In addition, serum phosphorus showed a U-shaped relationship with the prevalence of NAFLD. Stratified analyses identified significant factors influencing these relationships, suggesting that these factors are key players. We demonstrated the stability of the findings by multiple sensitivity analyses showing that these relationships were independent of important risk factors affecting blood calcium. Our results suggest that the impact of blood calcium on NAFLD may be altered by albumin, which requires that clinicians pay close attention to changes in serum calcium, as well as albumin, in people at risk for NAFLD. In addition, albumin-adjusted serum calcium may serve as a potential biomarker in the newer nomenclatures, i.e., SLD and MASLD.

Only three observational studies have explored the correlation between serum/blood calcium and NAFLD, while only one study has shown that serum phosphorus was associated with NAFLD. Shin et al. (17) showed that albumin-adjusted serum calcium was positively associated with ultrasound-diagnosed NAFLD in a cross-sectional Korean cohort, consistent with our findings. Yang et al. (15) included a cohort from China that showed that lower serum calcium was associated with an increased risk of NAFLD by a case-control approach. This is also consistent with the finding that serum calcium without adjustment for albumin was inversely associated with NAFLD in our study. Another case-control study from China similarly showed that blood calcium was inversely associated with NAFLD (16). These studies and ours similarly suggest that serum albumin adjustment may reverse the effect of serum calcium on NAFLD. Shin et al. (17) indicated that serum phosphorus was also positively associated with NAFLD, which differed from our study. Our results showed that serum phosphorus had a U-shaped relationship with the prevalence of NAFLD and was associated with the lowest prevalence of NAFLD at 3.8 mg/dL and not associated with NAFLD when it was greater than 3.8 mg/dL. Our data are on the one hand consistent with some of the previous findings and on the other hand indicate for the first time that serum calcium has an opposite trend in relation to NAFLD before and after adjustment for albumin. In addition, our RCS findings suggest that albumin-adjusted serum calcium and serum phosphorus levels are associated with NAFLD prevalence only prior to the inflection point (9.2 mg/dL and 3.8 mg/dL, respectively), which may suggest that lower blood calcium/phosphorus levels require more attention.

The association of serum calcium with the prevalence/incidence of several metabolic disorders has been extensively studied. A recent study combining the NHANES and Mendelian randomization study showed a significant positive association between serum calcium and type 2 diabetes, albeit without adjusting for serum albumin (27). The association of blood calcium with the risk of incident diabetes is controversial. A previous meta-analysis showed that serum total and albumin-adjusted calcium were positively correlated with incident diabetes (28), consistent with the findings of a retrospective cohort study (29). However, a subsequent study showed that albumin-adjusted serum calcium was unrelated to the incidence of diabetes (30). Serum calcium has also been shown to be involved in the development of metabolic syndrome (MetS). A large cross-sectional study showed a positive correlation between serum calcium and the development of MetS (31). Other cross-sectional studies have similarly suggested that serum calcium is positively associated with the development of MetS (32, 33), although a retrospective cohort study did not find a significant relationship (34).

The association of serum phosphorus with metabolic diseases has also been investigated in several studies. A cross-sectional study showed that either excessively low or high serum phosphorus was implicated in the development of MetS, similar to our findings (35). Therefore, it can be speculated that abnormalities in serum phosphorus levels, whether elevated or decreased, are associated with metabolic dysregulation. Low serum phosphorus may impair energy metabolism, whereas excess phosphorus may contribute to elevated blood pressure (36). These mechanisms may explain the U-shaped relationship between serum phosphorus and NAFLD (the hepatic manifestation of MetS) found in our study.

It remains unknown how albumin affects the correlation between serum calcium and NAFLD. We observed significantly lower serum albumin levels in NAFLD patients compared to participants without NAFLD in our baseline analysis. Serum albumin levels may serve as a predictor of impaired liver function in NAFLD, and the function of albumin is also compromised in NAFLD (37). Low serum albumin levels and impaired albumin binding capacity are associated with poor prognosis in NAFLD (38, 39). Thus, we found that regardless of whether serum calcium was adjusted using a low (4 g/dL) or high (4.4 g/dL) serum albumin reference value, albumin-adjusted serum calcium would always be higher at the population level than in participants without NAFLD due to the significant decline in serum albumin in the NAFLD population. Since most of the bound calcium in the blood is bound to albumin, high or low albumin levels can affect the measured serum total calcium level. Although albumin levels affect serum bound calcium levels, however, physiologically active ionized calcium levels remain stable. Therefore, abnormal albumin levels may lead to an overestimation or underestimation of ionized calcium levels. In our study, the NAFLD population showed either hyperalbuminemia or hypoalbuminemia compared to the “normal” population at different albumin reference values. Correcting serum total calcium levels for changes in albumin levels with or without NAFLD more accurately reflects the level of stabilized ionized calcium.

Circadian rhythm dysregulation has been implicated as a novel regulator in the pathogenesis of NAFLD and is involved in the modulation of hormonal and metabolic homeostasis (40–42). Notably, the regulation of serum calcium and phosphorus concentrations is also influenced by circadian rhythms (43, 44). Therefore, according to research by Gnocchi et al. (40, 42), we hypothesize that circadian clock misalignment of serum calcium/phosphorus concentrations may partially explain the association of serum calcium and phosphorus with NAFLD. In addition, thyroid dysfunction as an important pathophysiologic mechanism in NAFLD is also proposed to participate in the circadian rhythm of serum calcium/phosphorus (45). Thus, circadian rhythms may serve as the primary molecular machinery involved in NAFLD onset and progression by orchestrating thyroid function and serum calcium/phosphorus. However, given that no direct experimental evidence is currently available, future mechanistic studies are needed to confirm these conjectures.

We identified several factors that can significantly influence the correlation between serum calcium/phosphorus and NAFLD. We found that gender significantly influenced these correlations, and the associations were more pronounced among females compared to males. The exact mechanism is still not known, and previous studies have not reported these results. However, NAFLD has been shown to be a sexually dimorphic disease (46, 47). The prevalence in women is generally lower than in men, although it is higher in postmenopausal women. Thus, differences in sex hormones such as estrogen in men and women may influence the relationship between serum calcium/phosphorus and NAFLD, and future mechanistic studies are needed to unravel the underlying causes. In addition, serum albumin and calcium levels may also be affected by gender (48, 49).

We found that physical activity, eGFR, hypertension, and CVD were also important influences on albumin-adjusted serum calcium in relation to NAFLD. Physical activity may lead to an increase in serum total and ionized calcium by inducing a low-grade metabolic acidosis (50). We observed a significant association only in those with eGFR ≥60 ml/min/1.73m². The kidney is an important regulator of serum albumin and calcium, and our results suggest that impaired renal function confounds albumin-adjusted calcium effects. Hypertension significantly enhanced the effect of albumin-adjusted serum calcium on NAFLD. Several observational studies have shown that serum total calcium is positively associated with hypertension, even when adjusted for serum albumin (51, 52). However, we found an albumin-adjusted serum calcium effect on NAFLD only in the CVD-free population. Higher albumin-adjusted serum calcium may be associated with an increased risk of CVD (53). These results suggest that the correlation between albumin-adjusted calcium and NAFLD, although independent of these factors, is still being affected in some way. Future studies are needed to explore potential explanations.

Our results remain stable across multiple sensitivity analyses. Vitamin D can help with calcium absorption and deposition in the bones and is the primary vitamin for maintaining blood calcium and phosphorus balance. PTH is also an important hormone that controls blood calcium and phosphorus homeostasis in the body. Thus, our findings suggest that the correlation between serum calcium/phosphorus and NAFLD is independent of these important regulators. These effects were present even in those with normal BMD, suggesting that these associations may be independent of the influence of bone calcium. However, our additional investigation only found albumin-adjusted serum calcium to be associated with SLD and MASLD. We speculate that this may be caused by differences in diagnostic criteria for SLD and MASLD compared with NAFLD, as SLD additionally takes into account other possible causes of steatosis, whereas MASLD additionally takes into account metabolic disorders as a component. Given that studies on these newly named conditions are still scarce, future large-sample cohort studies are needed to confirm our findings and explore potential mechanisms.

Our study has some significant advantages. First, the nature of large sample and national representativeness confers good generalizability to our study. Our study was extensively adjusted for confounders, including important factors such as dietary intake, eGFR, vitamin D, and PTH, ensuring the reliability of the results. A comprehensive understanding into clinical practice has been provided by exploring differences in the role of serum calcium in response to albumin adjustment. However, our study has shortcomings. It is a cross-sectional study, and therefore causality could not be derived, and residual confounding factors may still exist. The diagnosis of NAFLD was based on noninvasive serologic biomarkers rather than imaging tests, which may have compromised accuracy. However, we obtained similar results in both FLI and USFLI, validating the stability of the results. In a future perspective, large-sample studies are needed to verify our findings.

## Conclusions

In a large population-based cross-sectional study, we found significant associations between serum calcium/phosphorus and NAFLD. These correlations are independent of significant influences and may be gender specific. Our study supports the potential role of serum calcium/phosphorus homeostasis in the pathophysiology of NAFLD and highlights the importance of focusing on serum calcium/phosphorus in clinical practice in populations at risk for NAFLD. Furthermore, our findings suggest that albumin-adjusted serum calcium may be clinically significant in SLD and MASLD.

## Data availability statement

The original contributions presented in the study are included in the article/**Supplementary Material**. Further inquiries can be directed to the corresponding author.

## Ethics statement

The studies involving humans were approved by the NCHS Ethics Review Board and no additional ethical review consent is required. All participants have provided written informed consent. The studies were conducted in accordance with the local legislation and institutional requirements. Written informed consent for participation in this study was provided by the participants’ legal guardians/next of kin.

## Author contributions

HQ: Data curation, Writing – original draft. BW: Formal analysis, Methodology, Writing – original draft. LZ: Conceptualization, Funding acquisition, Writing – review & editing.
