# Supplementary material for: Independent associations of serum calcium with or without albumin adjustment and serum phosphorus with nonalcoholic fatty liver disease: results from NHANES 1999-2018
Source: Front Endocrinol (Lausanne). 2024 Mar 5;15:1323990. doi: 10.3389/fendo.2024.1323990 (PMC10948406; doi:10.3389/fendo.2024.1323990)
Supplement: Supplementary file 1 [file Table_1.doc]

**Table S1**. Serum calcium, albumin-adjusted serum calcium, and serum phosphorus interquartile ranges.

|  | **Q1** | **Q2** | **Q3** | **Q4** |
| --- | --- | --- | --- | --- |
| **Serum calcium** |  |  |  |  |
| N | 4470 | 4349 | 6831 | 5250 |
| Value range | 7.400-9.100 | 9.200-9.300 | 9.400-9.600 | 9.700-12.700 |
| **Albumin-adjusted serum calcium** |  |  |  |  |
| N | 5221 | 4949 | 5364 | 5366 |
| Value range | 7.160-8.980 | 9.000-9.180 | 9.200-9.400 | 9.420-12.160 |
| **Serum phosphorus** |  |  |  |  |
| N | 4235 | 5751 | 4485 | 6429 |
| Value range | 1.500-3.200 | 3.300-3.600 | 3.700-3.900 | 4.000-8.900 |

**Table S2**. Baseline analysis based on serum calcium quartile groupings.

| **Variable** | **Total** | **Q1** | **Q2** | **Q3** | **Q4** | **P value** |
| --- | --- | --- | --- | --- | --- | --- |
| Age, year | 48.05±0.23 | 48.44±0.32 | 48.10±0.36 | 47.68±0.30 | 48.17±0.34 | 0.24 |
| PIR | 3.29±0.03 | 3.28±0.04 | 3.30±0.04 | 3.33±0.04 | 3.23±0.04 | 0.04 |
| Physical activity, Met/min-week | 3017.59±65.05 | 3389.65±105.17 | 3044.23±104.21 | 3073.99±101.14 | 2624.16± 96.67 | < 0.0001 |
| eGFR, ml/min/1.73m² | 92.53±0.31 | 94.29±0.45 | 93.57±0.50 | 92.49±0.37 | 90.35±0.46 | < 0.0001 |
| Calcium intake, mg/day | 956.70±6.04 | 923.47± 9.91 | 952.85±11.06 | 966.43± 9.17 | 973.70±10.41 | < 0.001 |
| Phosphorus intake, mg/day | 1380.36±6.55 | 1347.70±11.92 | 1378.74±12.52 | 1398.25±10.27 | 1384.42±11.71 | 0.01 |
| Serum phosphorus, mg/dl | 3.70±0.01 | 3.56±0.01 | 3.64±0.01 | 3.73±0.01 | 3.84±0.01 | < 0.0001 |
| Serum total calcium, mg/dl | 9.44±0.01 | 8.96±0.00 | 9.25±0.00 | 9.49±0.00 | 9.89±0.00 | < 0.0001 |
| Albumin-corrected serum total calcium, mg/dl | 9.19±0.01 | 8.89±0.01 | 9.08±0.01 | 9.22±0.00 | 9.50±0.01 | < 0.0001 |
| Albumin, g/dl | 4.30±0.00 | 4.08±0.01 | 4.22±0.01 | 4.34±0.01 | 4.48±0.01 | < 0.0001 |
| Sex |  |  |  |  |  | < 0.0001 |
| male | 10416(48.72) | 1956(41.34) | 2035(44.92) | 3645(52.11) | 2780(53.28) |  |
| female | 10484(51.28) | 2514(58.66) | 2314(55.08) | 3186(47.89) | 2470(46.72) |  |
| Race |  |  |  |  |  | < 0.0001 |
| Mexican American | 2917(5.73) | 840(7.93) | 649(6.19) | 903(5.36) | 525(4.06) |  |
| Non-Hispanic Black | 4062(9.40) | 778(8.95) | 802(8.97) | 1294(9.03) | 1188(10.59) |  |
| Non-Hispanic White | 10581(74.38) | 2039(71.32) | 2189(74.45) | 3515(74.80) | 2838(76.20) |  |
| Other Hispanic | 1449(4.38) | 341(4.87) | 300(4.14) | 487(4.59) | 321(3.89) |  |
| Other Race | 1891(6.12) | 472(6.93) | 409(6.25) | 632(6.21) | 378(5.25) |  |
| Marital Status |  |  |  |  |  | 0.02 |
| non-single | 13542(69.31) | 2986(70.47) | 2844(69.34) | 4420(70.26) | 3292(67.12) |  |
| single | 7358(30.69) | 1484(29.53) | 1505(30.66) | 2411(29.74) | 1958(32.88) |  |
| Education |  |  |  |  |  | 0.001 |
| <high school | 1658(3.59) | 421(4.01) | 367(3.78) | 513(3.46) | 357(3.25) |  |
| high school | 6884(29.79) | 1441(29.68) | 1346(27.33) | 2226(29.56) | 1871(32.19) |  |
| >high school | 12358(66.62) | 2608(66.31) | 2636(68.90) | 4092(66.98) | 3022(64.55) |  |
| Smoking |  |  |  |  |  | 0.002 |
| never | 12267(59.35) | 2622(58.75) | 2642(62.20) | 4021(59.35) | 2982(57.50) |  |
| former | 5534(26.24) | 1249(28.16) | 1088(23.98) | 1774(26.02) | 1423(26.83) |  |
| now | 3099(14.41) | 599(13.09) | 619(13.82) | 1036(14.63) | 845(15.67) |  |
| Diabetes |  |  |  |  |  | 0.69 |
| No | 17692(88.79) | 3768(88.96) | 3696(88.49) | 5838(89.14) | 4390(88.43) |  |
| Yes | 3208(11.21) | 702(11.04) | 653(11.51) | 993(10.86) | 860(11.57) |  |
| Hypertension |  |  |  |  |  | < 0.0001 |
| No | 12358(64.41) | 2777(67.49) | 2659(67.01) | 4077(64.75) | 2845(59.38) |  |
| Yes | 8542(35.59) | 1693(32.51) | 1690(32.99) | 2754(35.25) | 2405(40.62) |  |
| CVD |  |  |  |  |  | 0.64 |
| No | 18851(92.44) | 3993(92.09) | 3915(92.28) | 6218(92.81) | 4725(92.36) |  |
| Yes | 2049(7.56) | 477(7.91) | 434(7.72) | 613(7.19) | 525(7.64) |  |
| NAFLD |  |  |  |  |  | 0.02 |
| No | 11568(57.15) | 2350(54.85) | 2373(56.77) | 3813(57.15) | 3032(59.29) |  |
| Yes | 9332(42.85) | 2120(45.15) | 1976(43.23) | 3018(42.85) | 2218(40.71) |  |

Abbreviation: NAFLD, nonalcoholic fatty liver disease; PIR, family income to poverty; eGFR, estimated glomerular filtration rate. Continuous variables were expressed using mean ± standard error, while categorical variables were described using number (percentage).

**Table S3**. Baseline analysis based on albumin-corrected serum calcium quartile groupings.

| **Variable** | **Total** | **Q1** | **Q2** | **Q3** | **Q4** | **P value** |
| --- | --- | --- | --- | --- | --- | --- |
| Age, year | 48.05±0.23 | 45.41±0.33 | 47.01±0.35 | 48.37±0.34 | 51.81±0.35 | < 0.0001 |
| PIR | 3.29±0.03 | 3.38±0.04 | 3.36±0.04 | 3.28±0.04 | 3.10±0.04 | < 0.0001 |
| Physical activity, Met/min-week | 3017.59±65.05 | 3334.00±122.45 | 3231.76± 93.55 | 2921.45± 88.46 | 2533.16± 96.16 | < 0.0001 |
| eGFR, ml/min/1.73m² | 92.53±0.31 | 96.04±0.43 | 93.49±0.42 | 91.91±0.40 | 88.18±0.44 | < 0.0001 |
| Calcium intake, mg/day | 956.70±6.04 | 971.38± 9.37 | 983.58±10.20 | 938.89± 9.51 | 930.94±11.11 | < 0.0001 |
| Phosphorus intake, mg/day | 1380.36±6.55 | 1430.89±10.66 | 1413.41±10.83 | 1360.28±10.10 | 1309.49±11.89 | < 0.0001 |
| Serum phosphorus, mg/dl | 3.70±0.01 | 3.61±0.01 | 3.68±0.01 | 3.74±0.01 | 3.81±0.01 | < 0.0001 |
| Serum total calcium, mg/dl | 9.44±0.01 | 9.14±0.01 | 9.36±0.01 | 9.50±0.00 | 9.78±0.01 | < 0.0001 |
| Albumin-corrected serum total calcium, mg/dl | 9.19±0.01 | 8.81±0.00 | 9.09±0.00 | 9.29±0.00 | 9.64±0.00 | < 0.0001 |
| Albumin, g/dl | 4.30±0.00 | 4.42±0.01 | 4.33±0.01 | 4.26±0.01 | 4.18±0.01 | < 0.0001 |
| Sex |  |  |  |  |  | < 0.0001 |
| male | 10416(48.72) | 2942(55.14) | 2662(52.15) | 2649(48.09) | 2163(38.40) |  |
| female | 10484(51.28) | 2279(44.86) | 2287(47.85) | 2715(51.91) | 3203(61.60) |  |
| Race |  |  |  |  |  | < 0.0001 |
| Mexican American | 2917(5.73) | 1008(7.70) | 717(6.04) | 663(4.87) | 529(4.08) |  |
| Non-Hispanic Black | 4062(9.40) | 551(5.04) | 806(7.57) | 1159(10.47) | 1546(15.16) |  |
| Non-Hispanic White | 10581(74.38) | 2634(74.87) | 2547(75.47) | 2773(75.39) | 2627(71.51) |  |
| Other Hispanic | 1449(4.38) | 374(4.60) | 363(4.31) | 376(4.36) | 336(4.22) |  |
| Other Race | 1891(6.12) | 654(7.79) | 516(6.61) | 393(4.91) | 328(5.03) |  |
| Marital Status |  |  |  |  |  | < 0.0001 |
| non-single | 13542(69.31) | 3626(72.32) | 3314(71.27) | 3400(68.14) | 3202(65.08) |  |
| single | 7358(30.69) | 1595(27.68) | 1635(28.73) | 1964(31.86) | 2164(34.92) |  |
| Education |  |  |  |  |  | < 0.0001 |
| <high school | 1658(3.59) | 500(4.00) | 358(3.21) | 399(3.26) | 401(3.87) |  |
| high school | 6884(29.79) | 1540(26.57) | 1528(27.39) | 1817(31.23) | 1999(34.46) |  |
| >high school | 12358(66.62) | 3181(69.43) | 3063(69.40) | 3148(65.51) | 2966(61.67) |  |
| Smoke |  |  |  |  |  | < 0.001 |
| never | 12267(59.35) | 3154(60.91) | 2946(60.21) | 3162(59.81) | 3005(56.12) |  |
| former | 5534(26.24) | 1410(26.60) | 1272(25.74) | 1379(25.31) | 1473(27.39) |  |
| now | 3099(14.41) | 657(12.49) | 731(14.05) | 823(14.88) | 888(16.49) |  |
| Diabetes |  |  |  |  |  | < 0.0001 |
| No | 17692(88.79) | 4615(91.70) | 4327(90.76) | 4543(89.00) | 4207(83.11) |  |
| Yes | 3208(11.21) | 606(8.30) | 622(9.24) | 821(11.00) | 1159(16.89) |  |
| Hypertension |  |  |  |  |  | < 0.0001 |
| No | 12358(64.41) | 3538(72.26) | 3112(67.23) | 3122(64.06) | 2586(52.80) |  |
| Yes | 8542(35.59) | 1683(27.74) | 1837(32.77) | 2242(35.94) | 2780(47.20) |  |
| CVD |  |  |  |  |  | < 0.0001 |
| No | 18851(92.44) | 4791(94.14) | 4523(93.35) | 4845(92.41) | 4692(89.55) |  |
| Yes | 2049(7.56) | 430(5.86) | 426(6.65) | 519(7.59) | 674(10.45) |  |
| NAFLD |  |  |  |  |  | < 0.0001 |
| No | 11568(57.15) | 3199(63.54) | 2812(58.24) | 2869(54.75) | 2688(51.32) |  |
| Yes | 9332(42.85) | 2022(36.46) | 2137(41.76) | 2495(45.25) | 2678(48.68) |  |

Abbreviation: NAFLD, nonalcoholic fatty liver disease; PIR, family income to poverty; eGFR, estimated glomerular filtration rate. Continuous variables were expressed using mean ± standard error, while categorical variables were described using number (percentage).

**Table S4**. Baseline analysis based on serum phosphorus quartile groupings.

| **Variable** | **Total** | **Q1** | **Q2** | **Q3** | **Q4** | **P value** |
| --- | --- | --- | --- | --- | --- | --- |
| Age, year | 48.05±0.23 | 48.44±0.34 | 48.56±0.30 | 48.06±0.34 | 47.35±0.31 | 0.004 |
| PIR | 3.29±0.03 | 3.25±0.04 | 3.29±0.04 | 3.31±0.04 | 3.29±0.04 | 0.47 |
| Physical activity, Met/min-week | 3017.59±65.05 | 3312.37±113.78 | 3.29±0.04 | 2820.05±107.64 | 2854.59± 82.33 | < 0.001 |
| eGFR,ml/min/1.73m² | 92.53±0.31 | 92.78±0.41 | 93.01±0.38 | 92.55±0.49 | 91.95±0.43 | 0.15 |
| Calcium intake, mg/day | 956.70±6.04 | 948.41±11.87 | 939.86± 8.70 | 955.38±11.84 | 977.48± 9.53 | 0.02 |
| Phosphorus intake, mg/day | 1380.36±6.55 | 1391.41±12.16 | 1373.24± 9.65 | 1373.08±12.37 | 1384.58±10.43 | 0.51 |
| Serum phosphorus, mg/dl | 3.70±0.01 | 2.95±0.01 | 3.46±0.00 | 3.80±0.00 | 4.33±0.01 | < 0.0001 |
| Serum total calcium, mg/dl | 9.44±0.01 | 9.35±0.01 | 9.41±0.01 | 9.44±0.01 | 9.51±0.01 | < 0.0001 |
| Albumin-corrected serum total calcium, mg/dl | 9.19±0.01 | 9.13±0.01 | 9.17±0.01 | 9.20±0.01 | 9.25±0.01 | < 0.0001 |
| Albumin, g/dl | 4.30±0.00 | 4.28±0.01 | 4.29±0.01 | 4.29±0.01 | 4.33±0.01 | < 0.0001 |
| Sex |  |  |  |  |  | < 0.0001 |
| male | 10416(48.72) | 2661(61.85) | 3087(52.39) | 2050(44.39) | 2618(40.21) |  |
| female | 10484(51.28) | 1574(38.15) | 2664(47.61) | 2435(55.61) | 3811(59.79) |  |
| Race |  |  |  |  |  | 0.08 |
| Mexican American | 2917(5.73) | 661(6.35) | 793(5.66) | 658(6.18) | 805(5.08) |  |
| Non-Hispanic Black | 4062(9.40) | 842(9.47) | 1152(9.68) | 864(9.41) | 1204(9.11) |  |
| Non-Hispanic White | 10581(74.38) | 2132(74.58) | 2904(74.13) | 2236(73.90) | 3309(74.79) |  |
| Other Hispanic | 1449(4.38) | 275(4.06) | 374(4.04) | 319(4.70) | 481(4.65) |  |
| Other Race | 1891(6.12) | 325(5.54) | 528(6.49) | 408(5.81) | 630(6.37) |  |
| Marital Status |  |  |  |  |  | 0.05 |
| non-single | 13542(69.31) | 2848(71.44) | 3738(68.80) | 2888(69.41) | 4068(68.36) |  |
| single | 7358(30.69) | 1387(28.56) | 2013(31.20) | 1597(30.59) | 2361(31.64) |  |
| Education |  |  |  |  |  | < 0.001 |
| <high school | 1658(3.59) | 382(3.79) | 465(3.55) | 359(3.73) | 452(3.39) |  |
| high school | 6884(29.79) | 1511(31.96) | 1947(31.16) | 1434(29.14) | 1992(27.68) |  |
| >high school | 12358(66.62) | 2342(64.25) | 3339(65.29) | 2692(67.13) | 3985(68.93) |  |
| Smoke |  |  |  |  |  | < 0.0001 |
| never | 12267(59.35) | 2404(58.82) | 3279(56.98) | 2724(61.49) | 3860(60.28) |  |
| former | 5534(26.24) | 1233(27.55) | 1655(29.14) | 1124(24.52) | 1522(24.07) |  |
| now | 3099(14.41) | 598(13.63) | 817(13.88) | 637(13.99) | 1047(15.65) |  |
| Diabetes |  |  |  |  |  | 0.14 |
| No | 17692(88.79) | 3509(88.26) | 4873(88.30) | 3846(89.95) | 5464(88.75) |  |
| Yes | 3208(11.21) | 726(11.74) | 878(11.70) | 639(10.05) | 965(11.25) |  |
| Hypertension |  |  |  |  |  | 0.17 |
| No | 12358(64.41) | 2426(63.55) | 3337(63.34) | 2697(65.53) | 3898(65.13) |  |
| Yes | 8542(35.59) | 1809(36.45) | 2414(36.66) | 1788(34.47) | 2531(34.87) |  |
| CVD |  |  |  |  |  | < 0.001 |
| No | 18851(92.44) | 3773(91.18) | 5150(91.77) | 4085(93.47) | 5843(93.11) |  |
| Yes | 2049(7.56) | 462(8.82) | 601(8.23) | 400(6.53) | 586(6.89) |  |
| NAFLD |  |  |  |  |  | < 0.0001 |
| No | 11568(57.15) | 2115(51.51) | 3232(57.36) | 2587(60.49) | 3634(58.23) |  |
| Yes | 9332(42.85) | 2120(48.49) | 2519(42.64) | 1898(39.51) | 2795(41.77) |  |

Abbreviation: NAFLD, nonalcoholic fatty liver disease; PIR, family income to poverty; eGFR, estimated glomerular filtration rate. Continuous variables were expressed using mean ± standard error, while categorical variables were described using number (percentage).

**Table S5**. Piecewise regression analysis.

|  | OR (95%CI) P-value |
| --- | --- |
| Albumin-adjusted serum calcium |  |
| ≤9.2 mg/dL | 2.12 (1.58, 2.85) **<0.0001** |
| >9.2 mg/dL | 1.12 (0.90, 1.41) 0.3144 |
| Serum phosphorus |  |
| ≤3.8 mg/dL | 0.73 (0.64, 0.83) **<0.0001** |
| >3.8 mg/dL | 1.14 (0.97, 1.34) 0.1183 |

Abbreviation: OR, odds ratio.

**Table S6**. Sensitivity analysis using the USFLI to diagnose NAFLD.

|  | **Crude Model**  **OR (95%CI)**  **P-value** | **Model 1**  **OR (95%CI)**  **P-value** | **Model 2**  **OR (95%CI)**  **P-value** | **Model 3**  **OR (95%CI)**  **P-value** |
| --- | --- | --- | --- | --- |
| Serum calcium | 0.95 (0.81, 1.13) 0.5668 | 0.88 (0.73, 1.04) 0.1418 | 0.74 (0.62, 0.88) 0.0010 | 0.74 (0.62, 0.89) **0.0016** |
| Serum calcium Quartile |  |  |  |  |
| Q1 | Ref. | Ref. | Ref. | Ref. |
| Q2 | 0.96 (0.80, 1.15) 0.6640 | 0.95 (0.78, 1.15) 0.5821 | 0.92 (0.75, 1.12) 0.3920 | 0.92 (0.75, 1.12) 0.3999 |
| Q3 | 1.02 (0.87, 1.21) 0.7868 | 0.99 (0.83, 1.17) 0.8658 | 0.93 (0.78, 1.10) 0.3960 | 0.93 (0.78, 1.11) 0.4181 |
| Q4 | 0.93 (0.79, 1.11) 0.4288 | 0.87 (0.72, 1.04) 0.1345 | 0.75 (0.62, 0.90) 0.0023 | 0.75 (0.63, 0.91) **0.0033** |
| P for trend | 0.98 (0.93, 1.04) 0.5435 | 0.96 (0.91, 1.02) 0.1566 | 0.91 (0.87, 0.97) 0.0020 | 0.92 (0.87, 0.97) **0.0032** |
| Albumin-adjusted serum calcium | 1.90 (1.60, 2.25) <0.0001 | 2.02 (1.67, 2.44) <0.0001 | 1.61 (1.32, 1.95) <0.0001 | 1.64 (1.34, 1.99) **<0.0001** |
| Albumin-adjusted serum calcium Quartile |  |  |  |  |
| Q1 | Ref. | Ref. | Ref. | Ref. |
| Q2 | 1.30 (1.14, 1.50) 0.0002 | 1.32 (1.14, 1.54) 0.0003 | 1.31 (1.11, 1.53) 0.0015 | 1.31 (1.11, 1.54) **0.0014** |
| Q3 | 1.43 (1.21, 1.70) <0.0001 | 1.50 (1.25, 1.80) <0.0001 | 1.46 (1.21, 1.77) 0.0001 | 1.48 (1.22, 1.79) **0.0001** |
| Q4 | 1.67 (1.41, 1.98) <0.0001 | 1.75 (1.45, 2.12) <0.0001 | 1.46 (1.20, 1.77) 0.0002 | 1.47 (1.21, 1.79) **0.0002** |
| P for trend | 1.18 (1.12, 1.24) <0.0001 | 1.20 (1.13, 1.27) <0.0001 | 1.13 (1.07, 1.20) 0.0001 | 1.14 (1.07, 1.21) **0.0001** |
| Serum phosphorus | 0.76 (0.69, 0.84) <0.0001 | 0.89 (0.80, 0.99) 0.0381 | 0.82 (0.73, 0.92) 0.0012 | 0.82 (0.73, 0.93) **0.0015** |
| Serum phosphorus Quartile |  |  |  |  |
| Q1 | Ref. | Ref. | Ref. | Ref. |
| Q2 | 0.82 (0.70, 0.97) 0.0215 | 0.88 (0.74, 1.05) 0.1556 | 0.86 (0.72, 1.04) 0.1278 | 0.86 (0.71, 1.04) 0.1206 |
| Q3 | 0.69 (0.60, 0.80) <0.0001 | 0.78 (0.67, 0.91) 0.0024 | 0.74 (0.63, 0.88) 0.0006 | 0.74 (0.63, 0.87) **0.0006** |
| Q4 | 0.74 (0.64, 0.85) <0.0001 | 0.92 (0.79, 1.07) 0.2946 | 0.84 (0.71, 0.99) 0.0419 | 0.84 (0.71, 0.99) **0.0426** |
| P for trend | <0.0001 | 0.1698 | 0.0157 | **0.0162** |

Abbreviation: NAFLD, nonalcoholic fatty liver disease; OR, odds ratio; eGFR, estimated glomerular filtration rate. The crude model did not adjust for any covariates. Model 1 adjusted for age, sex, race, education, PIR, and marital status; model 2 additionally adjusted for smoking, diabetes, hypertension, CVD, and physical activity in addition to model 1; and model 3 additionally adjusted for eGFR and dietary calcium/phosphorus intake in addition to model 2.

**Table S7**. Sensitivity analysis with additional adjustment for vitamin D based on model 3.

|  | Serum phosphorus OR (95%CI) P-value | Serum calcium  OR (95%CI) P-value | Albumin-adjusted serum calcium OR (95%CI) P-value |
| --- | --- | --- | --- |
| Continuous | 0.95 (0.89, 1.01) 0.1101 | 0.70 (0.62, 0.79) **<0.0001** | 1.56 (1.37, 1.78) **<0.0001** |
| Quartile |  |  |  |
| Q1 | Ref. | Ref. | Ref. |
| Q2 | 0.82 (0.73, 0.92) **0.0010** | 0.90 (0.78, 1.03) 0.1155 | 1.26 (1.11, 1.43) **0.0005** |
| Q3 | 0.77 (0.68, 0.87) **<0.0001** | 0.83 (0.73, 0.94) **0.0057** | 1.42 (1.27, 1.58) **<0.0001** |
| Q4 | 0.87 (0.78, 0.98) **0.0211** | 0.69 (0.60, 0.80) **<0.0001** | 1.49 (1.31, 1.69) **<0.0001** |
| P for trend | 0.0878 | **<0.0001** | **<0.0001** |

Abbreviation: OR, odds ratio.

**Table S8**. Additional adjustment of PTH for sensitivity analysis based on model 3.

|  | Serum phosphorus OR (95%CI) P-value | Serum calcium  OR (95%CI) P-value | Albumin-adjusted serum calcium OR (95%CI) P-value |
| --- | --- | --- | --- |
| Continuous | 1.07 (0.94, 1.21) 0.3094 | 0.65 (0.53, 0.78) **<0.0001** | 1.38 (1.11, 1.70) **0.0034** |
| Quartile |  |  |  |
| Q1 | Ref. | Ref. | Ref. |
| Q2 | 0.76 (0.61, 0.93) **0.0089** | 0.92 (0.72, 1.16) 0.4703 | 1.32 (1.04, 1.68) **0.0219** |
| Q3 | 0.76 (0.61, 0.94) **0.0122** | 0.80 (0.65, 1.00) **0.0483** | 1.39 (1.11, 1.75) **0.0040** |
| Q4 | 0.97 (0.79, 1.18) 0.7381 | 0.67 (0.54, 0.83) **0.0003** | 1.50 (1.20, 1.88) **0.0004** |
| P for trend | 0.4595 | **<0.0001** | **0.0012** |

Abbreviation: PTH, parathyroid hormone; OR, odds ratio.

**Table S9**. Sensitivity analysis in a population with normal BMD in model 3.

|  | **OR (95%CI) P-value** |
| --- | --- |
| Serum calcium | 0.72 (0.62, 0.85) **<0.0001** |
| Calcium Quartile |  |
| Q1 | Ref. |
| Q2 | 0.76 (0.63, 0.91) **0.0025** |
| Q3 | 0.76 (0.64, 0.89) **0.0010** |
| Q4 | 0.71 (0.60, 0.85) **0.0002** |
| P for trend | **0.0005** |
| Albumin-adjusted serum calcium | 1.41 (1.18, 1.70) **0.0002** |
| Albumin-adjusted serum calcium Quartile |  |
| Q1 | Ref. |
| Q2 | 1.21 (1.02, 1.43) **0.0322** |
| Q3 | 1.20 (1.02, 1.42) **0.0306** |
| Q4 | 1.37 (1.15, 1.63) **0.0004** |
| P for trend | **0.0009** |
| Serum phosphorus | 0.98 (0.90, 1.07) 0.6307 |
| Serum phosphorus Quartile |  |
| Q1 | Ref. |
| Q2 | 0.80 (0.70, 0.92) **0.0017** |
| Q3 | 0.85 (0.73, 0.98) **0.0265** |
| Q4 | 0.90 (0.79, 1.03) 0.1399 |
| P for trend | 0.5580 |

Abbreviation: BMD, bone mineral density; OR, odds ratio.

**Table S10**. Serum calcium correction using another serum albumin reference value (4.4 g/dL).

|  | **Crude Model**  **OR (95%CI)**  **P-value** | **Model 1**  **OR (95%CI)**  **P-value** | **Model 2**  **OR (95%CI)**  **P-value** | **Model 3**  **OR (95%CI)**  **P-value** |
| --- | --- | --- | --- | --- |
| Adjust-Calcium (4.4) | 1.78 (1.60, 1.98) <0.0001 | 1.81 (1.61, 2.03) <0.0001 | 1.57 (1.40, 1.77) <0.0001 | 1.59 (1.41, 1.79) **<0.0001** |
| Adjust-Calcium Quartile |  |  |  |  |
| Q1 | Ref. | Ref. | Ref. | Ref. |
| Q2 | 1.25 (1.12, 1.39) 0.0001 | 1.26 (1.13, 1.41) 0.0001 | 1.24 (1.11, 1.39) 0.0003 | 1.25 (1.11, 1.40) **0.0002** |
| Q3 | 1.44 (1.30, 1.59) <0.0001 | 1.46 (1.31, 1.62) <0.0001 | 1.42 (1.28, 1.57) <0.0001 | 1.43 (1.29, 1.58) **<0.0001** |
| Q4 | 1.65 (1.48, 1.84) <0.0001 | 1.68 (1.50, 1.89) <0.0001 | 1.48 (1.32, 1.67) <0.0001 | 1.50 (1.33, 1.68) **<0.0001** |
| P for trend | <0.0001 | <0.0001 | <0.0001 | **<0.0001** |

Table S11. Association of blood calcium/phosphorus with SLD and MASLD.

|  | **MASLD** | **SLD** |
| --- | --- | --- |
| Adjusted**-**Calcium | | |
| Adjusted | 1.60 (1.13, 2.26) 0.0025 | 1.39 (1.12,1.74) 0.0067 |
| Adjusted |  |  |
| Q1 | Ref. | Ref. |
| Q2 | 1.09 (0.86, 1.38) 0.4920 | 0.86 (0.70, 1.07) 0.1889 |
| Q3 | 1.24 (0.88, 1.74) 0.2282 | 1.06 (0.84, 1.34) 0.6019 |
| Q4 | 1.44 (1.21, 1.28) 0.0002 | 1.31 (1.05, 1.63) 0.0281 |
| P for trend | 0.0437 | 0.0098 |
| **Calcium** | | |
| Calcium | 1.07 (0.75, 1.53) 0.7228 | 0.83 (0.63, 1.09) 0.1975 |
| Calcium |  |  |
| Q1 | Ref. | Ref. |
| Q2 | 0.87 (0.64, 1.19) 0.3996 | 0.83 (0.65, 1.06) 0.1450 |
| Q3 | 1.07 (0.78, 1.47) 0.3997 | 0.91 (0.70, 1.18) 0.4840 |
| Q4 | 0.98 (0.69, 1.40) 0.9077 | 0.78 (0.77, 1.02) 0.0828 |
| P for trend | 0.6819 | 0.2212 |
| **Phosphorus** | | |
| Phosphorus | 0.94 (0.79, 1.12) 0.4550 | 0.94 (0.80, 1.11) 0.4794 |
| Phosphorus |  |  |
| Q1 | Ref. | Ref. |
| Q2 | 0.98 (0.71, 1.37) 0.9197 | 0.80 (0.58, 1.11) 0.2053 |
| Q3 | 0.84 (0.65, 1.08) 0.1574 | 0.83 (0.62, 1.12) 0.2357 |
| Q4 | 0.98 (0.79, 1.21) 0.8211 | 0.86 (0.70, 1.05) 0.1558 |
| P for trend | 0.5270 | 0.2511 |

Adjusted for age, sex, race, education, PIR, and marital status, smoking, diabetes, hypertension, CVD, physical activity, eGFR, and dietary calcium/phosphorus intake. SLD, steatotic liver disease; MASLD, metabolic dysfunction-associated steatotic liver disease.

Abbreviation: NAFLD, nonalcoholic fatty liver disease; OR, odds ratio. The crude model did not adjust for any covariates. Model 1 adjusted for age, sex, race, education, PIR, and marital status; model 2 additionally adjusted for smoking, diabetes, hypertension, CVD, and physical activity in addition to model 1; and model 3 additionally adjusted for eGFR and dietary calcium/phosphorus intake in addition to model 2.
